# Are Core Stability Tests Related to Single Leg Squat Performance in Active Females?

**DOI:** 10.3390/ijerph18115548

**Published:** 2021-05-22

**Authors:** Paloma Guillén-Rogel, David Barbado, Cristina Franco-Escudero, Cristina San Emeterio, Pedro J. Marín

**Affiliations:** 1 Laboratory of Physiology, Faculty of Health Sciences, Miguel de Cervantes European University, 47012 Valladolid, Spain; pguillen@uemc.es (P.G.-R.); cristinafrancoescudero@gmail.com (C.F.-E.); cristinasanemeteriogarcia@msn.com (C.S.E.); 2 Sport Research Centre, Miguel Hernández University, 03202 Elche, Spain; dbarbado@umh.es; 3 Development Research, CYMO Research Institute, 47140 Valladolid, Spain

**Keywords:** accelerometer, assessment, lumbopelvic-hip complex, mobile technology

## Abstract

Core stability (CS) deficits can have a significant impact on lower limb function. The aim of this study was to investigate the relationship between two dynamic core exercise assessments and dynamic knee valgus during single-leg squats. In total, 20 physically active female students participated in this study. The OCTOcore smartphone application assesses CS during two dynamic exercise tests, the partial range single-leg deadlift (SLD) test and the bird-dog (BD) test. A two-dimensional assessment of a single-leg squat test was used to quantify participants’ hip frontal angle (HFA_SLS_) and knee frontal plane projection angle (FPPA_SLS_). Ankle dorsiflexion was evaluated through the weight-bearing dorsiflexion test. The correlational analyses indicated that the HFA_SLS_ was significantly related to the partial range single-leg deadlift test (*r* = 0.314, *p* < 0.05) and ankle dorsiflexion (*r* = 0.322, *p* < 0.05). The results showed a significant difference (*p* < 0.05) in the CS test between cases categorised as dynamic knee valgus (>10°) and normal (≤10°). The CS deficit may influence the neuromuscular control of the lumbopelvic-hip complex during single-leg movements. The link between CS and kinematic factors related to knee injuries was only observed when CS was measured in the SLD test but not in the BD test.

## 1. Introduction

Effective knee injury prevention programs require an extensive understanding of the aetiological factors and physical factors, and any underlying mechanism that can trigger an injury [1,2,3].

Among the most important modifiable factors with respect to knee injuries, especially in female sport populations, is dynamic knee valgus (DKV). DKV can be considered a lower limb movement pattern characterised by excessive medial movement during weight-bearing movements [4]. It is observed during single-leg movement tasks [5] and has been extensively described as an injury mechanism for non-contact injuries in females, such as anterior cruciate ligament (ACL) injury [6,7], patellofemoral pain [8], or chronic hip joint pain [4]. The knee frontal plane projection angle (FPPA) as an index of dynamic valgus was estimated by measuring the angle between a drawn line bisecting the thigh and another line bisecting the lower leg [9]. This angle captures the two-dimensional motion of the tibia and femur but not the pelvis motion. Thus, the knee and the hip angles may be a more ample representation of the whole lower-extremity movement pattern than the knee FPPA alone [10]. Additionally, ankle kinematics in weight bearing activities should not be ignored in the investigation of lower limb biomechanics; there is evidence of the effect of the ankle dorsiflexion (DF_ANK_) restriction on DKV in a single-leg squat (SLS) [5].

A growing body of research suggests [11,12] that core stability (CS) deficits can have a significant impact on lower limb function [13]. Based on this idea, poor CS is currently considered a crucial factor in the development of lower extremity injuries, which are usually solely attributed to lower limb malfunction [14,15]. Specifically, cross-sectional studies have concluded that during cutting manoeuvres, a higher trunk tilting away from the direction of the cut is linked to larger knee abduction peak moments [16,17], which induce higher ACL strain [18,19]. Prospective studies [20,21] have also shown an association between different factors related to the neuromuscular control of the core (i.e., displacement of the trunk before an external perturbation, proprioceptive control of the position of the trunk, etc.) and sports knee injuries in females for three years following data collection. Finally, two intervention studies argued that core training programs can reduce knee loading [22,23]. All these results suggest that athletes’ CS should be periodically checked to identify an impaired status, which can increase the risk of knee injuries. However, the control of athletes’ CS status in the professional field presents several limitations, as it not only hinders the determination of those who are at risk of injury but also obstructs the understanding of the effect of core training programs. 

Typically, laboratory CS testing applies sophisticated equipment, such as 3D tracking systems, force plates, and strain gauges [24,25]. Although these devices are highly valid and reliable, their costs make them unsuitable for quantifying CS outside the lab. Conversely, despite the low cost and easy application of field tests, most of them have exhibited several methodological limitations such as low reliability, reduced sensitivity, and limited validity for measuring CS [25].

Among the different laboratory instruments used to quantify postural control, accelerometers have become increasingly cheap and accessible [26,27]. Today, sensors, such as accelerometers, gyroscopes, and compasses are commonly embedded in several daily use devices, such as smartphones, thereby implying a sensitivity comparable to that of traditional biomechanical equipment [28,29]. Recently, two studies have confirmed that smartphone accelerometers can be used to obtain a reliable quantification of CS during isometric and quasi-isometric tasks [25,30], which creates a new opportunity to explore the relationship between CS and DKV in field settings.

This study performed two novel tests (bird-dog and single-leg deadlift) to assess core stability deficits outside the laboratory with one novel measurement (an accelerometer embedded in a smartphone) and also investigated whether they could detect movements that could be a risk factor for knee injury. In order to achieve this aim, the relationship between the core stability and DKV during a single-leg squat in females by these methods was be examined. Since a decreased DF_ANK_ range of motion (ROM) during weight-bearing tasks limits the ability to control the body, especially in the frontal plane, the potential influence of DF_ANK_ on CS and DKV was also explored.

## 2. Materials and Methods

### 2.1. Participants

An a-priori power analysis was conducted to estimate the sample size. G*Power software (G*Power 3.1.9.6 Kiel University, Kiel, Germany) estimated a sample size of 18 subjects (significance level = 0.05; required power = 0.80; correlation among repeated measures = 0.30).

Overall, 20 physically active female students (age: 22.4 ± 7.65 years; body weight: 60.3 ± 6.7 kg; height: 162.2 ± 6.7 cm) were recruited from the academic community for this study. Only female participants were approached because they present a higher knee injury prevalence [31] associated with a specific lower extremity kinematic behaviour [32,33,34]. To be included in the study, participants should not have suffered musculoskeletal injuries in the last six months. All participants were recreationally active. They were physically active, performing 1–2 h of moderate physical activity 1–2 days per week. Subjects were not allowed to participate in this study if (i) they suffered from any cardiovascular, respiratory, abdominal, neurological, musculoskeletal, or other chronic diseases; (ii) they presented symptoms that could affect the musculoskeletal system; or (iii) they did not exercise for longer than 150 min per week. The study was conducted according to the guidelines of the Declaration of Helsinki and approved by the Institutional Review Board CyMO Research Institute (1.200.550).

### 2.2. Procedures

Participants completed three laboratory sessions in this study (2 familiarisation sessions and 1 test session) one week apart from each other. All the participants were requested to refrain from exercising 48 h prior to testing to reduce the potential influence of fatigue on the physical tests. 

During the testing session, participants performed the following tests with each leg in a randomized order: weight-bearing dorsiflexion test, the bird-dog test, the partial range single-leg deadlift core stability test and the single-leg squat test. All sessions were performed at the same time of day to minimize the effect of circadian rhythms.

#### 2.2.1. Weight-Bearing Dorsiflexion Test

The maximal DF_ANK_ was evaluated with the LegMotion^®^ system (Check your Motion^®^, Albacete, Spain) in a weight-bearing position [35,36]. During testing, participants were barefoot and kept their hands on their hips. Participants placed one foot in the middle of the longitudinal line of the LegMotion^®^ platform just behind the transversal line. The uninvolved foot was placed off the platform with toes close to the platform edge. In this position, the participants flexed their knee as much as possible, trying to touch a marker placed just in front of the patella without raising their ankle heel. DF_ANK_ is defined as the maximum distance (cm) achieved. Three trials were allowed with each ankle (e.g., left and right) with 10 s of passive recovery between trials. The third value from each ankle was used in the subsequent analyses.

#### 2.2.2. Bird-Dog (BD) Test

**Figure 1:**
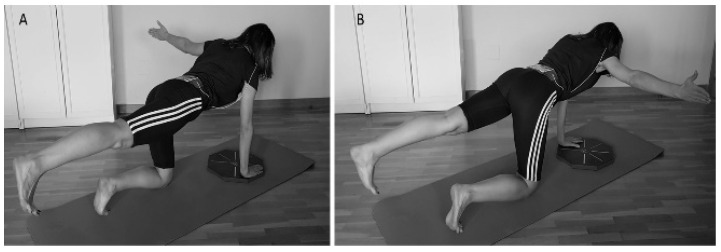
Bird-dog (BD) test. Variation of bird-dog exercise test raising the left (**A**) and (**B**) right arm to the side following the direction of the OctoBalance^®^ line.

The participants performed the “bird-dog” with the OCTOcore application (Check your Motion^®^, Albacete, Spain) and following a previously described procedure [30]. Specifically, the BD is a core stability exercise in which the opposite upper and lower extremities are raised in a quadruped position. Participants were requested to reduce their trunk and pelvic motion as much as possible while keeping the lumbar spine and pelvis in a neutral position. During the BD, they had to raise one arm and the opposite leg following a “tick” sound emitted by the OCTOcore app at a cadence of 20 bpm. The application indicated which arm and leg should be raised in a random order using “grey” or “green” colours. When the grey colour appeared, the participants had to raise their right arm and left leg (Figure 1). Opposite limbs had to be used for a green colour signal. Each exercise (grey or green) was performed for 30 repetitions as a familiarisation trial. After a three-minute break, participants performed 50 repetitions. Participants were subsequently asked to perform the exercise at a controlled speed during the three seconds that each repetition lasted. 

#### 2.2.3. Partial Range Single-Leg Deadlift Core Stability Test (SLD)

**Figure 2:**
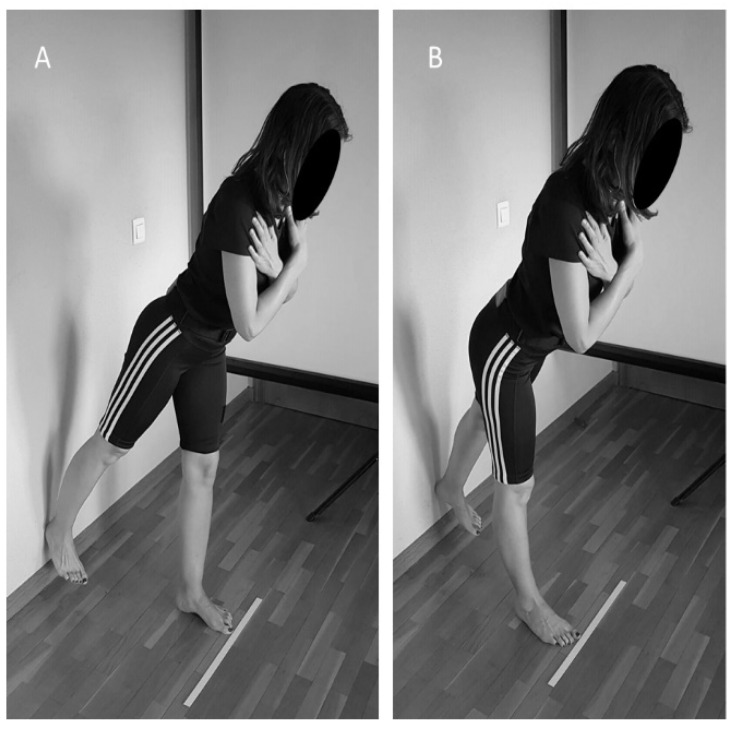
Partial range single leg deadlift test with the right heel (**A**) and the left heel (**B**) touching the wall.

The participants’ core stability was assessed while they performed a partial-range single-leg deadlift (SLD). The core stability during the SLD was quantified, recording lower back linear accelerations from a 3-axis accelerometer embedded in a smartphone (iPhone^®^ model 6, Apple Inc., Cupertino, CA, USA) using the OCTOcore application [30] (Check your Motion^®^, Albacete, Spain) from which earth gravity was excluded. An adjustable belt was used to place the smartphone on the midline of each participant’s back between the iliac crests and the fourth lumbar vertebra. In the SLD, participants had to touch the wall with their right or left heel, tilting the trunk forward while keeping their trunk in the neutral position and their legs straight (Figure 2) according to a “left” or “right” order given by the OCTOcore app. After performing the movement, participants had to return to the starting position and await the next order. They were asked to perform an SLD at a moderate velocity (three seconds per repetition) while constantly looking forward. Participants performed 30 warm-up repetitions and, after a three-minute rest performed 50 evaluation repetitions. 

#### 2.2.4. Single-Leg Squat Test (SLS)

A two-dimensional assessment of a single-leg squat test was used to quantify participants’ FPPA_SLS_ and hip frontal angle (HFA_SLS_). A digital camera (FDR-AX33, Sony, Tokio, Japan) was placed on a tripod 3 m in front of the participant at the level of their knees. Pictures were taken throughout the entire movement, covering participants’ bodies from their feet to their trunk [37]. Furthermore, markers were placed on the following landmarks: anterior superior iliac spine (ASIS), patella, tibial tuberosity and centre of the talocrural joint. To reduce the bias caused by shoe differences on limb movement, participants removed their shoes before testing. Participants were requested to perform a single-leg squat as far down as comfortably possible for four seconds [8], keeping their trunk upright, their arms crossed over their chest, and flexing their knee to at least 60°, which was visually confirmed by a researcher [4,38]. The non-stance leg was flexed at the knee to 90°. Participants performed 10 practice trials with each limb to become comfortable with the task. The knee flexion angle was checked during practice trials using a goniometer. After a 3-min rest, each participant performed 5 repetitions with each limb. During prior testing, a researcher provided a visual demonstration of the test.

Videos from each repetition were collected. The recording was repeated if the participant lost balance during the movement or did not reach the 60° angle desired. We defined when a participant (i) placed the untested limb on the floor or (ii) moved the stance limb (e.g., sliding, hopping, or twisting). 

### 2.3. Analysis

#### 2.3.1. Data Analysis and Reduction

Each participant’s dynamic knee valgus was assessed from each recorded video using the software Kinovea 0.8.24 (Kinovea, Burdeaux, France) [39]. Specifically, the starting and ending positions of each SLD were analysed. The frame before the participant’s tested knee started flexing was considered the starting position. The frame in which the knee reached its maximal flexion was considered the ending position. 

The hip frontal plane was the angle between both ASIS and a line from the ipsilateral ASIS to the centre of the patella [40]. The FPPA was estimated by measuring the angle between a drawn line bisecting the thigh and another line from the knee joint to the ankle [10,41]. Positive values represented a knee motion with the knee towards the body midline, and negative values represented a knee motion away from the midline. The mean knee FPPA was calculated from 5 valid trials. The data from all five trials were ensemble-averaged for each leg.

#### 2.3.2. Statistical Analysis

Descriptive statistics (mean ± SD) were calculated for all variables in both the familiarization and test sessions. The normality of the data was examined using a Kolmogorov–Smirnov statistical test. To analyse the between-session absolute reliability of the SLD and BD, the standard error of measurement (SEM) was calculated as the standard deviation of the difference between the best result of the familiarisation session and the testing session divided by √2. This SEM method was adopted to avoid the influence of sample heterogeneity and to reduce the effect of systematic error (e.g., learning effect). SEM values were expressed as a percentage of the mean score, which facilitates the extrapolation of the results to other individuals and reliability comparisons with other protocols. The relative reliability of the different measures was analysed using the ICC_2,1_, calculating 95% confidence limits (95% CL). The ICC values were categorised as follows: excellent (0.90 to 1.00), high (0.70 to 0.89), moderate (0.50 to 0.69), and low (<0.50) [42]. One-way repeated-measures ANOVAs were performed to assess the repetition effect, with the session as the within-subject factor (familiarisation and testing sessions). Additionally, the effect size statistic, Cohen’s d, was analysed to determine the magnitude of the effect independent of sample size.

Pearson correlations were performed to analyse the potential relationship between core stability and DKV. To reduce the potential influence of learning effects on the results, further correlational analyses were performed using the participants’ best score obtained in the familiarisation or testing sessions. Finally, based on the participants’ DV_SLS_ scores obtained during the single-leg squat test, each leg´s performance was categorised as DKV (>10°) or normal (≤10°) [9]. Following this, Mann–Whitney U tests for independent measures were used to assess the core stability and ankle ROM differences of legs classified as DKV and non-dynamic knee valgus. All statistical analyses were conducted using SPSS (Version 22.0, IBM, Armonk, NY, USA). Statistical significance was established at *p* < 0.05.

## 3. Results

The core stability assessment conducted in the BD and SLD using the OCTOcore application indicated high relative reliability scores (Table 1), with ICC values ranging from 0.71 to 0.84. Similarly, both the BD and SLD exhibited SEM scores lower than 20% (Table 1), which is considered acceptable for posturographic parameters. Specially, the SLD exhibited higher SEM scores (9.7 ≤ SEM ≤ 11.34) than the BD (13.0 ≤ SEM ≤ 17.3).

The correlational analyses (Table 2) showed that the only parameter associated with FPPA_SLS_ is DF_ANK_ (*r* = −0.337). The HFA_SLS_ was significantly related to the SLD (*r* = 0.314) and DFANK (*r* = 0.322).

Finally, the Mann–Whiney U test showed significant differences in the SLD between cases categorised as dynamic knee valgus (*n* = 35) and normal (*n* = 5) (Table 3). There were no significant differences observed for the DF_ANK_ or BD (Table 3). 

## 4. Discussion

The first aim of this study was to assess whether core stability can be assessed through an accelerometer embedded in a smartphone during two different exercises that impose a challenge with regard to the control of core structures. The second and predominant aim was to analyse whether core stability is related to DKV in physically active young women.

Our initial results confirmed that the OCTOcore application is a reliable tool for assessing core stability during partial single-leg death lift and bird-dog tests. These findings are in accordance with the conclusions of previous works, which have shown that accelerometers embedded in smartphones or iPods are useful tools for quantifying postural control in a broad range of tasks, such as isometric core stability exercises [25] and upright stance balance tasks [25,43]. The most important implication of the reliability finding of this study is that our results reinforce that core stability can be reliably quantified in quasi-static movements [30], which creates a new opportunity for assessing this ability in other ecological tasks.

With respect to the correlational results, the most relevant finding is that higher SLD scores are related to higher hip adduction during single-leg squats, which has been highlighted as a relevant factor influencing the frontal plane motion and load of the knee [44]. Specifically, a greater lateral inclination of the trunk far from the stance limb reflected by a larger pelvic tilt creates and increases valgus joint moment. Therefore, our results support that core stability deficit may influence the neuromuscular control of the trunk during single-leg movements, thereby reinforcing the potential link between poor lateral trunk stability and the risk of knee injury [20]. However, our investigations linking the core stability measures with knee motion in the frontal plane exhibited mixed results. On the one hand, although our correlational results indicated a clear relationship between HFA_SLS_ and FPPA_SLS_ (*r* = 0.849), no direct relationships were observed between core stability assessed during the BD and SLS and the degree of knee valgus during the single-leg squat exercise. Nevertheless, on the other hand, our between-group comparison analysis showed that leg performance categorised as dynamic knee valgus (>10°) exhibited worse core stability during the SLD compared to leg performance categorised as normal (≤10°). From the authors’ point of view, the lack of dynamic valgus in participants (DKV ≤ 10º) showing improved core stability scores in the SLD suggests that the relationship between core stability and DKV is not linear. The larger DKV displayed during the SLS is not significantly affected by a greater or poorer neuromuscular control of the core, which would reinforce other factors, such as hip strength, landing technique, or anthropometry, as the most determinant ones influencing knee valgus. Nevertheless, in female athletes who did not exhibit extremely large DKV scores, core stability could play a relevant role in modifying knee mechanics. Thus, in this case, poor neuromuscular control may induce subtle changes in lower limb function, which would be determinant of female athletes showing DKV scores above the limit of risk for knee injuries (DKV > 10°). 

The present research is one of the first studies in the field using an inexpensive and reliable tool to quantify core stability that can be used in professional settings. However, it presents some limitations. First, the data of this study are limited to healthy and physically active young females, which limits the generalizability of the results. Although a sample of 20 participants has been considered a sufficient sample, a much larger sample would be desirable for minimizing any random changes ocurring in the measurements. 

Secondly, this study did not test the potential link and interaction between core stability and other elements that the literature has shown to impact knee motion (e.g., hip strength). Furthermore, patterns of activity among muscles were not assessed, making it difficult to specifically identify which muscles are most related to which deficits. Thus, future studies should include individuals with different spinal conditions, musculoskeletal risk factors, ages, and physical activity levels (including types of physical activity). Finally, future studies should compare an intervention group to a control group to further test and solidify this relationship. 

## 5. Conclusions

The present study reveals that the OCTOcore application is a tool for assessing core stability during partial SLD and BD tests. The core stability deficit may influence the neuromuscular control of the lumbopelvic-hip complex during single-leg movements. These results indicate that the link between core stability and kinematic factors related to knee injuries was only observed when core stability was measured in the SLD and not in the BD. The SLD test by OCTOcore application is an easily performed, widely available and well tolerated test for assessing the core stability of physically active female with DKV in everyday clinical practice.

## Figures and Tables

**Table 1:** Reliability scores for the bird-dog (BD) and the partial range single-leg deadlift core stability test (SLD) in healthy young women (*n* = 20).

Task	Familiarisation Session ^a^	First Session ^a^	*F*	*p*	*d*	SEM		ICC_3,1_^c^
						Units ^b^	Percentage ^c^	
BD_R_	8.5 ± 2.8	8.5 ± 2.5	0.01	0.92	−0.02	1.1	13.0 (9.8–19.6)	0.84 (0.62–0.94)
BD_L_	9.2 ± 3.2	9.9 ± 3.3	1.58	0.22	0.19	1.6	17.3 (13.1–25.2)	0.77 (0.50–0.90)
SLD_R_	13.0 ± 3.0	12.3 ± 2.6	1.46	0.24	−0.22	1.2	9.7 (7.3–14.4)	0.81 (0.57–0.92)
SLD_L_	13.0 ± 2.8	12.3 ± 2.3	2.12	0.16	−0.23	1.4	11.4 (8.7–16.7)	0.71 (0.40–0.87)

Repeated measures analysis of variance. ^a^ Data are presented with mean ± SD; ^b^ data are presented with mean; ^c^ data are presented with mean (95% CI). Units for all tests: mm/s^−2^. Abbreviations: CI: confidence interval at 95%; *d*: effect size; BDL: bird-dog rising the left leg and the right arm; BDR: bird-dog rising the right leg and the left arm; SLDL: partial range single-leg deadlift core stability test rising the left leg; SLDR: partial range single-leg deadlift core stability test rising the right leg.

**Table 2:** Correlations of the participants’ core stability assessed during the bird-dog (BD) and the partial range single-leg deadlift test (SLD) and their ankle range of motion (DFANK).

Variables	BD	SLD	DF_ANK_	FPPA_SLS_	HFA_SLS_
BD		0.139	−0.389 *	0.043	0.039
SLD			−0.316 *	0.234	0.314 *
DF_ANK_				−0.337 *	0.322 *
FPPA_SLS_					0.849 **
HFA_SLS_					

HFA_SLS_: hip frontal angle. FPPA_SLS_: knee frontal angle. **: Correlation is significant at the 0.01 level (2-tailed). *: Correlation is significant at the 0.05 level (2-tailed).

**Table 3:** Differences in ankle range of motion (DFANK) and core stability assessed in the bird-dog test (BD) and the partial range single-leg deadlift test between those legs categorised as dynamic knee valgus (DKV > 10°; *n* = 35) and normal (DKV ≤ 10°; *n* = 5).

Variables	DKV ^a^	Normal ^a^	*U Score*	*p*
DF_ANK_ (cm)	11.5 (10.5–12.1)	13.5 (10.7–14.5)	57.5	0.228
BD (mm/s^−2^)	8.1 (7.2–9.1)	8.0 (5.5–10.2)	84.0	0.905
SLD (mm/s^−2^)	12.0 (11.3–12.9)	9.5 (6.6–12.6)	1135.5	0.047

Mann–Whitney U test for independent measures. ^a^ Data are presented as median (confidence interval at 95%).

## Data Availability

The data presented in this study are available on request from the corresponding author.
